# Concomitant mitral valve repair and resynchronization therapy

**DOI:** 10.1186/1749-8090-8-S1-O271

**Published:** 2013-09-11

**Authors:** S Borovic, V Ristic, L Angelkov, Z Vukajlovic, P Dabic, B Djukanovic

**Affiliations:** 1 Dedinje Cardiovascular Institute, Belgrade, Serbia

## Background

Functional mitral regurgitation (MR) affects 90% of cardiac resynchronization therapy (CRT) candidates, with moderate–severe/severe MR being present in 35%. The purpose was to assess the outcome of CRT candidates with severe MR undergoing concomitant mitral valve repair and resynchronization therapy.

## Methods

A case series of 3 consecutive patients underwent concomitant mitral valve repair and resynchronization therapy between September 2011 and January 2012 at our institution. Prospectively recorded preoperative, intraoperative, and postoperative data were retrospectively screened for in-hospital mortality, adverse postoperative events and functional and echocardiographic changes. Patients were reevaluated at 6 and 12 months.

## Results

There was no in-hospital mortality and major adverse postoperative events. End-systolic LV volumes decreased by >20% compared with baseline at 6-month and 12-month follow-up in first and second patient. Third patient was not considered echocardiographic responder (no reduction in LV volume). First and second patient improved >10% the distance covered in the 6-minute walking test at follow-up. Positive clinical response was absent in third patient. At 6-month and 12-month MR was 2 and 3+ respectively in third patient. No MR recurrence was observed in remaining patients.

## Conclusions

CRT in patients with no residual MR yields improved reverse remodeling, response to resynchronization, functional status and survival (clinical and ECHO responders). Concomitant mitral valve repair and resynchronization therapy is feasible, safe, with acceptable results.

